# Longitudinal evolution of diffusion metrics after left hemisphere ischaemic stroke

**DOI:** 10.1093/braincomms/fcad313

**Published:** 2023-11-20

**Authors:** Johémie Boucher, Karine Marcotte, Christophe Bedetti, Bérengère Houzé, Maxime Descoteaux, Amélie Brisebois, Alberto Osa García, Elizabeth Rochon, Carol Leonard, Alex Desautels, Simona M Brambati

**Affiliations:** Department of Psychology, Centre de recherche de l’Institut Universitaire de Gériatrie de Montréal, Montréal, QC H3W 1W5, Canada; Département de psychologie, Faculté des arts et des sciences, Université de Montréal, Montréal, QC H3C 3J7, Canada; Centre de recherche du Centre intégré universitaire de santé et de services sociaux du Nord-de-l’Île-de-Montréal, Montréal, QC H4J 1C5, Canada; École d’orthophonie et d’audiologie, Faculté de médecine, Université de Montréal, Montréal, QC H3N 1X7, Canada; Department of Psychology, Centre de recherche de l’Institut Universitaire de Gériatrie de Montréal, Montréal, QC H3W 1W5, Canada; Département de psychologie, Faculté des arts et des sciences, Université de Montréal, Montréal, QC H3C 3J7, Canada; Department of Psychology, Centre de recherche de l’Institut Universitaire de Gériatrie de Montréal, Montréal, QC H3W 1W5, Canada; Département de psychologie, Faculté des arts et des sciences, Université de Montréal, Montréal, QC H3C 3J7, Canada; Département d’informatique, Faculté des sciences, Université de Sherbrooke, Sherbrooke, QC J1K 2X9, Canada; Centre de recherche du Centre intégré universitaire de santé et de services sociaux du Nord-de-l’Île-de-Montréal, Montréal, QC H4J 1C5, Canada; École d’orthophonie et d’audiologie, Faculté de médecine, Université de Montréal, Montréal, QC H3N 1X7, Canada; Centre de recherche du Centre intégré universitaire de santé et de services sociaux du Nord-de-l’Île-de-Montréal, Montréal, QC H4J 1C5, Canada; École d’orthophonie et d’audiologie, Faculté de médecine, Université de Montréal, Montréal, QC H3N 1X7, Canada; Department of Speech-Language Pathology, Temerty Faculty of Medicine, University of Toronto, Toronto, ON M5S 1A8, Canada; KITE Research Institute, Toronto Rehab, University Health Network, Toronto, ON M5G 2A2, Canada; Heart and Stroke Foundation, Canadian Partnership for Stroke Recovery, Ottawa, ON K1G 5Z3, Canada; Rehabilitation Sciences Institute, University of Toronto, Toronto, ON M5G 1V7, Canada; School of Rehabilitation Sciences, University of Ottawa, Ottawa, ON K1N 6N5, Canada; Centre de recherche du Centre intégré universitaire de santé et de services sociaux du Nord-de-l’Île-de-Montréal, Montréal, QC H4J 1C5, Canada; Département des neurosciences, Faculté de médecine, Université de Montréal, Montréal, QC H3C 3J7, Canada; Department of Psychology, Centre de recherche de l’Institut Universitaire de Gériatrie de Montréal, Montréal, QC H3W 1W5, Canada; Département de psychologie, Faculté des arts et des sciences, Université de Montréal, Montréal, QC H3C 3J7, Canada

**Keywords:** white matter, stroke, diffusion magnetic resonance imaging metrics, lesional tissue, perilesional tissue

## Abstract

White matter is often severely affected after human ischaemic stroke. While animal studies have suggested that various factors may contribute to white matter structural damage after ischaemic stroke, the characterization of damaging processes to the affected hemisphere after human stroke remains poorly understood. Thus, the present study aims to thoroughly describe the longitudinal pattern of evolution of diffusion magnetic resonance imaging metrics in different parts of the ipsilesional white matter after stroke. We acquired diffusion and anatomical images in 17 patients who had suffered from a single left hemisphere ischaemic stroke, at 24–72 h, 8–14 days and 6 months post-stroke. For each patient, we created three regions of interest: (i) the white matter lesion; (ii) the perilesional white matter; and (iii) the remaining white matter of the left hemisphere. We extracted diffusion metrics (fractional anisotropy, mean, axial and radial diffusivities) for each region and conducted two-way repeated measures ANOVAs with stage post-stroke (acute, subacute and chronic) × regions of interest (white matter lesion, perilesional white matter and remaining white matter). Fractional anisotropy values stayed consistent across time-points, with significantly lower values in the white matter lesion compared to the perilesional white matter and remaining white matter tissue. Fractional anisotropy values of the perilesional white matter were also significantly lower than that of the remaining white matter. Mean, axial and radial diffusivities in the white matter lesion were all decreased in the acute stage compared to perilesional white matter and remaining white matter, but significantly increased in both the subacute and chronic stages. Significant increases in mean and radial diffusivities in the perilesional white matter were seen in the later stages of stroke. Our findings suggest that various physiological processes are at play in the acute, subacute and chronic stages following ischaemic stroke, with the infarct territory and perilesional white matter affected by ischaemia at different rates and to different extents throughout the stroke recovery stages. The examination of multiple diffusivity metrics may inform us about the mechanisms occurring at different time-points, i.e. focal swelling, axonal damage or myelin loss.

## Teaser

Boucher *et al*.’s findings suggest that examination of multiple diffusivity metrics may inform us about various physiological processes occurring within the lesional and perilesional white matter in the acute, subacute and chronic stages post-stroke, such as focal swelling, axonal damage or myelin loss.

## Graphical Abstract

**Graphical Abstract:**
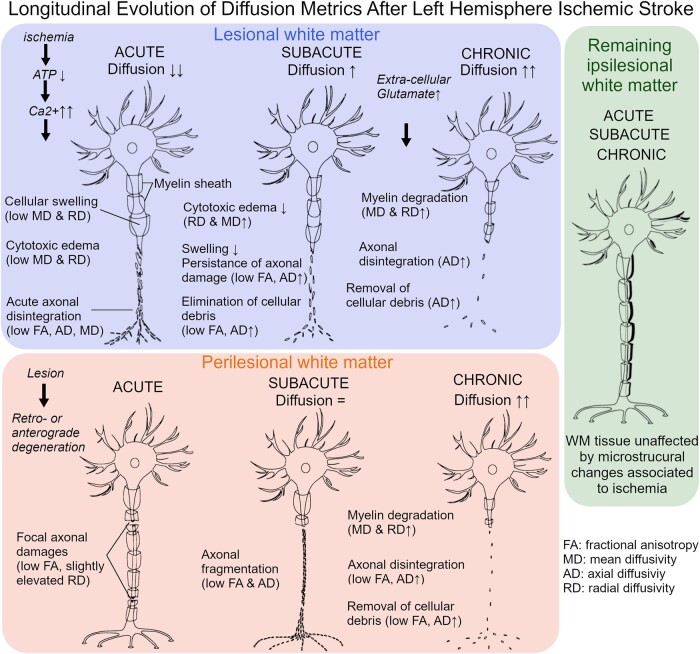

## Introduction

White matter (WM) is affected in most cases of human ischaemic stroke, with WM accounting for half of the lesion volume on average.^1^ Moreover, recent literature highlights the independent contribution of WM injury to neurological dysfunction after stroke.^1,2^ However, mechanisms of WM damage after ischaemic stroke, while distinctly different, have received far less attention than those of grey matter damage. The main explanation for this omission is that the brain of rodents, which are the most often used in animal stroke studies, has much less WM than human brains. Therefore, there has been a tendency to falsely believe that grey matter is more vulnerable to ischaemia than WM tissue^1,3^ and that the protection of neuron cell bodies alone is sufficient to promote brain recovery after stroke.^3^ Indeed, studies have shown that WM has lower blood flow than grey matter under normal conditions and that it receives less collateral circulation, making it particularly vulnerable to ischaemia.^1,3^ However, the pathophysiological mechanisms of WM damage after human stroke remain poorly understood.

Nonetheless, a few animal studies focusing on WM damage after stroke have helped develop models describing various mechanisms contributing to structural damages to lesional and perilesional tissue after ischaemic stroke, including both axonal and myelin disintegration. According to the most commonly proposed animal models,^1,3^ very early after stroke, hypoxia, ischaemia or glucose deprivation leads to energy reduction and loss of adenosine triphosphate (ATP) in the affected WM. Then, failure of Na^+^/K^+^ATPase leads to Na^+^ channel activation followed by Ca^2+^ entering the axons, causing a loss of ionic gradient reversibly altering the action potentials.^3^ Subsequent accumulations of toxic levels of calcium prompt molecular cascades leading to the degradation of the axonal cytoskeleton and organelles, focal axonal swelling and finally the disruption of axonal integrity.^3^ A parallel effect of energy depravation is the release of glutamate into the extracellular space from axons, astrocytes and oligodendrocytes, causing activation of ionotropic AMPA/Kainate receptors located on glial cells. Prolonged glutamate receptor activation then triggers excitotoxic damage to oligodendrocytes (i.e. myelin-producing cells) and oligodendrocyte progenitor cells, causing myelin damage.^1^ In sum, WM is highly vulnerable to ischaemia and is often severely damaged following stroke. Animal studies suggest that various factors may contribute to WM structural damage after ischaemic stroke, but the characterization of damaging processes affecting WM after human stroke remains poorly understood. Until recent years, proper techniques to investigate WM damages *in vivo* were lacking, but the development of diffusion magnetic resonance imaging (dMRI) has provided new tools to address this issue.

dMRI is a non-invasive technique that has been used to display the normal architecture of WM in humans, as well as the changes associated with brain injury.^1^ As water diffusion is constrained by the presence and the orientation of biological barriers,^4^ water in WM usually moves more easily along the axonal tract, while myelination restricts water movement perpendicular to the fasciculation.^5^ In general, studies have shown that structural modifications induced by stroke (e.g. axonal damage, swelling processes and demyelination) can significantly alter the characteristics of tissue water diffusion, making dMRI very suitable for the longitudinal assessment of WM damage after human stroke.^6^ Indeed, dMRI can be particularly useful as it allows the extraction of the microstructural characteristics of the tissue directly damaged by the stroke, and also of surrounding or more distant WM tissue. The most frequently reported diffusion measure of WM is fractional anisotropy (FA), followed by mean diffusivity (MD).^7^ FA quantifies the degree of directional dependence (anisotropy) of diffusion, while MD reflects the average magnitude of water molecules diffusion within a voxel.

Whereas both increased and reduced FA in the infarct and surrounding WM have been reported in the acute stage of human stroke, most studies indicate decreased FA in the later stages. For instance, several studies reported low FA values in the infarct site^8–10^ or perilesional WM^7,11–13^ in the early stages of stroke. Doughty *et al*.^14^ also found lower mean FA in the ipsilesional corticospinal tract within 80 h after ischaemic stroke onset, while Pinter *et al*.^7^ showed that FA in the WM of the lesioned hemisphere was reduced 24–72 h after ischaemic infarction in the territory of the middle cerebral artery. Studies in rodents found similar results, with reduced FA in most of the infarct site at 3 days post-stroke.^15^ However, there have also been reports of preserved or even increased FA early after stroke in the infarct site^16,17^ and surrounding WM.^18^ In fact, several human and animal studies convergently report significantly reduced FA in the chronic stage post-stroke in the perilesional or, more generally, in the ipsilesional WM.^7,8,13,19–21^ Decreased FA in the chronic stage is thought to reflect a permanent disruption of WM structural integrity e.g. due to axonal or myelin loss.^19^ This being said, some findings from animal^15^ and human studies^22^ reveal that the initial reductions of FA may be followed by normalization or enhancing of FA in the ischaemic perilesional tissue, suggesting that initial damages may be followed by a structural reorganization of surrounding WM in the later stages of stroke.

Regarding MD, most studies reported a decrease early after stroke, followed by a subsequent normalization and elevation in the subacute and chronic stages, respectively. Very early after cerebral ischaemia, cytotoxic oedema (i.e. shifting of water from the extracellular to the intracellular space) occurs, creating ‘beading’ of axons and dendrites and hereby hindering the movement of water molecules, causing MD to decrease in the ischaemic infarct territory^9,10,16^ and in ipsilesional surrounding WM.^12,23^ Pinter *et al*.,^7^ however, did not find significant reductions of MD in various regions of the ipsilesional WM 24–72 h following ischaemic stroke. This initial phase is usually followed by significant MD increases in the infarct territory^9,10^ and the perilesional and/or ipsilesional hemisphere,^7,8,20,21^ which may occur as soon as 7 days post-stroke and persist into the chronic stage. At this stage, prolonged energy depravation in the affected tissue is thought to cause the rupture of cell membranes (due to toxic intracellular accumulations of calcium) and/or demyelination processes (due to excitotoxic damage to oligodendrocytes and oligodendrocyte progenitor cells),^1^ increasing the extracellular space ^18^ and thereby permitting a larger displacement of water molecules for the same diffusion time (MD increases). Thus, diminished FA and elevated MD have been associated with loss of WM structural integrity chronically post-stroke, but the exact timeline regarding changes occurring between the acute and chronic stages remains ambiguous. Furthermore, while FA and MD are sensitive to microstructural WM changes, they are not specific to the type of change that occurs.^24,25^ For instance, low FA and high MD cannot help us to distinguish mechanisms of axonal disruption from those of myelin degeneration.^7^

Some studies have started to report measurements of the radial and axial diffusivities, which may demonstrate more specific relationships to white matter pathology. Radial diffusivity (RD) measures diffusivity perpendicular to the axons while axial diffusivity (AD) measures diffusivity parallel to the principal direction of the tensor, i.e. along the length of the axon. In general, RD is assumed to reflect myelin integrity, as some studies have confirmed increased RD in dysmyelination and demyelination.^26,27^ A few studies, however, have instead observed decreased AD or FA with demyelination.^28^ Usually, reductions in AD have been associated with axonal morphological changes.^1^

In the acute stage of stroke, the affected WM is often characterized by reduced values of AD with either normal or altered RD values, depending on the study. This pattern of diffusion is thought to reflect early axonal membrane disintegration creating barriers to water longitudinal diffusion and reducing AD in the infarct territory^10^ and surrounding white matter^11–13,23^ within the first days after stroke. Reduced RD that occurs in some studies may reflect acute cell swelling within the infarct territory in the early stages of stroke.^18,19^ Initial decreases in AD and/or RD are usually followed by progressive increases in the affected WM. Indeed, normal or higher AD and RD values have been noted in the subacute and/or chronic stages in the lesion core^9,10,29^ and in ipsilesional WM tracts, more generally.^13,21^ This pattern of diffusion has been associated with demyelination, causing RD increases, and removal of cellular debris resulting in re-establishment or increase in diffusion in the longitudinal direction (AD increases).

Although the association between dMRI metrics and physiological metrics is not completely established, available literature shows the importance of reporting the pattern of changes various metrics in stroke patients, in the lesional and perilesional WM tissue, to allow a better understanding of the evolution of the disease. However, most studies have failed to report changes from the acute to the chronic stage. In addition, these studies have often focused on distinct dMRI metrics, usually FA or MD, without giving a more global portrait combining different metrics. Finally, most studies have investigated WM of entire hemispheres or entire WM tracts, without looking at specific regions of interest (ROIs) relative to the lesion site (lesional, perilesional and ipsilesional WM excluding the lesion, etc.), which could account for the inconsistencies found in the existing literature. Thus, the objective of the present study is to characterize the pattern of dMRI metrics reporting all diffusivities in the WM involved in the original stroke lesion, the perilesional WM and the remaining ipsilesional WM at acute, subacute and chronic stages following stroke.

Thus, the objective of the present study is to characterize the evolution of all diffusivity metrics at different stages post-stroke in the WM involved in the original stroke lesion, the perilesional WM and the remaining ipsilesional WM. To this aim, for each post-stroke participant included in the study, we traced the WM lesion, perilesional WM and the remaining ipsilesional WM based on acute (24–72 h post-stroke) MRI images. From each of these regions, we then extracted and compared all diffusivity metrics based on dMRI images obtained at acute, subacute (between 8 and 14 days post-stroke) and chronic (∼6 months post-stroke) stages.

## Methods

### Participants

Seventeen participants, including eight female and nine male, were recruited from the stroke unit of ‘Hôpital du Sacré-Coeur de Montréal’, and were evaluated between May 2015 and August 2018. In order to include a more homogeneous group of patients with similar stroke aetiology, only right-handed participants who had suffered a first single ischaemic stroke in the middle cerebral artery of the left hemisphere were recruited. They were assessed at three time-points: the acute stage (24–72 h post-stroke); the subacute stage (between 8 and 14 days post-stroke); and the chronic stage (∼6 months post-stroke). Exclusion criteria included: history of major psychiatric disorders, learning disabilities, previous traumatic brain injury, previous intracranial surgery, previous stroke damaging the left and/or right hemisphere, uncorrected visual and/or hearing problems, haemorrhagic stroke, sub-thalamic stroke and/or other neurological conditions. Sociodemographic data are presented in Table 1. First language was French for all participants. The study was approved by the ethics review board of the ‘Centre intégré universitaire de santé et de services sociaux du Nord-de-l’Ile-de Montréal’ (Project #MP-32-2018-1478), and written informed consent was obtained from all participants.

**Table 1:** Sociodemographic characteristics and mean lesion volume data

*n*	17
Female/male ratio	8/9
Age	70.53 (±12.24)
Education	11.88 (±4.14)
Lesion volume (ml)	21 402.71 (±21 012.25)
WM lesion volume (ml)	8436 (±8641.87)

### MRI data acquisition

T_1_-weighted images were acquired with a Skyra 3T scanner (Siemens Healthcare, USA) at the Radiology Department of the Hôpital du Sacré-Coeur de Montréal. High resolution 3D T_1_-weighted images (TR = 2200 ms, TE = 2.96 ms, TI = 900 ms, FOV = 250 mm, voxel size = 1 × 1 × 1 mm^3^, matrix = 256 × 256, 192 slices, flip angle = 8°) were obtained using a Magnetization Prepared Rapid Gradient Echo (MPRAGE) sequence. Participants also underwent a dMRI acquisition protocol. Diffusion weighted images were acquired according to the following parameters: TR = 11 000 ms, TE = 86 ms, field of view = 230 mm, voxel resolution = 2 × 2 × 2 mm^3^, flip angle = 90°, bandwidth = 1700, EPI factor = 67, 68 sagittal slices covering the entire brain including the brainstem, with one T_2_-weighted image (*b* = 0 s/mm^2^) and 64 images with non-collinear diffusion directions with a *b*-value of 1000 s/mm^2^ in a posterior-anterior acquisition, and two T_2_-weighted images with *b* = 0 s/mm^2^, one being a posterior-anterior acquisition, the other an anterior-posterior acquisition (time of acquisition = 12 min and 30 s).

### Diffusion weighted imaging and T_1_-weighted image pre-processing

First, T_1_-weighted images were automatically processed with the longitudinal pipeline in FreeSurfer to create an unbiased within-subject template space ^30^ based on the three T_1_-weighted longitudinal images. Second, diffusion weighted imaging (DWI)-weighted images were pre-processed through several processing steps, including noise correction with the Marchenko–Pastur principal component analysis method using Mrtrix3,^31^ correction for subject movement, eddy-currents and susceptibility-induced distortions with AP-PA images using the commands eddy and topup of the FSL package,^32^ correction for N4 bias using the ANTs package,^33^ computation of the DTI metrics (fractional anisotropy [FA], mean diffusivity [MD], axial diffusivity [AD] and radial diffusivities [RD]) using DIPY,^34^ registration of T_1_-weighted images on the *b* = 0 mm^2^/s and the FA images using non-linear SyN from ANTs.^33^ All these steps were encapsulated in a fully automated and reproductible pipeline TractoFlow^35^ using the technologies of Nextflow^36^ and Singularity^37^ to run softwares in a fixed container with known software versions, thereby increasing the reproductibility of the analysis. In addition, Tractoflow is adapted for high performance computers, allowing more efficient analysis runtime.^35^ We used the Atlas Based Segmentation profile of TractoFlow version 2.3.0 recommended to run on pathological data^35^ with the Freesurfer anatomical images in the within-subject template space.

### Lesion demarcation and analysis

A semi-automated demarcation of each brain lesion in acute phase was performed for each participants using ‘Clusterize’^38^http://www.medizin.uni-tuebingen.de/kinder/en/research/neuroimaging/software/. This SPM toolbox (http://www.medizin.uni-tuebingen.de/kinder/en/research/neuroimaging/software/) has previously shown to have a good reliability in acute lesion demarcation in post-stroke patients.^39^ We used this toolbox with SPM12, running under Matlab (The Mathworks, Inc., Natick, MA).

Clusterize computed hypo-intensities clusters of voxels on MD maps (set with default parameters). Then, cluster(s)-of-interest corresponding to the lesion were manually selected and adjusted to fit the lesion in each slice by a study team member (B.H.). Second, each lesion file was counter-verified and adjusted (if needed) with MI-brain software (Imeka Solutions Inc.), with the help of MD maps and b0 DWI maps by two experienced in lesion delineation team members (B.H. and S.M.B.). Both raters were blind to participant’s identity.

Lesions were then realigned to the within-subject longitudinal space.^30^ For each patient, we created three ROIs based on the lesion images realigned in the within-subject longitudinal space:

1. The WM lesion, which consisted of the outlined lesion overlaid on a WM mask;
2. The perilesional WM (pWM), which was obtained for each participant by first creating a peri-infarct mask by dilating the infarct 8 mm using the scil_dilate_labels.py script (https://github.com/scilus/scilpy) and subtracting the initial infarct from the dilated infarct. To account for partial lesion volume, the peri-infarct area between 0 and 3 mm beyond the initial infarct rim was also subtracted, giving a thickness of 5 mm for this mask. The peri-infarct mask was then overlaid on a WM mask to create the pWM ROI; and
3. The remaining left hemisphere WM (rWM) was obtained by subtracting the lesion, the peri-infarct mask, and partial lesion volume boundary from a WM mask of the left hemisphere.

For each ROI, we extracted relevant dMRI metrics, i.e. FA, MD, AD and RD. WM lesion size was also computed.

### Statistical analyses

We conducted separate two-way repeated measures ANOVAs to analyse the effect of post-stroke stage (acute, subacute, chronic)×ROI (WM lesion, pWM, rWM) on each dMRI metric (FA, MD, AD and RD). Then, for *post hoc* analyses, we conducted multiple comparison analysis with Bonferroni correction.

## Results

**Figure 1:**
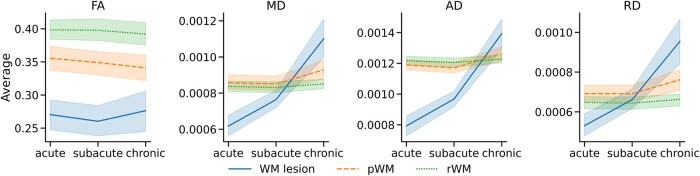
**Evolution of the diffusion metrics means (FA, MD, AD and RD) across time-points in the lesional, perilesional and remaining WM (*n* = 17).** WM lesion is in solid line, pWM in dashed line and rWM in dotted line. The upper and lower lines display the 95% confidence interval.

Table 2 reports the mean values of each diffusion metrics (FA, MD, AD, RD) at the three time-points (acute, subacute, chronic) and in the three ROIs (WM lesion, pWM, rWM). Tables 3 and 4 report the statistical results for ANOVAs, with Bonferroni correction for multiple comparisons. Figure 1 displays the evolution of the four mean diffusion metrics (FA, MD, AD, RD) at the three time-points and in the three ROIs (WM lesion, pWM, rWM).

**Table 2:** Mean and standard deviation of diffusion metrics in each ROI across the post-stroke stages (*n* = 17)

	Stages	WM lesion		pWM		rWM	
Metrics	Post-stroke	Mean	SD	Mean	SD	Mean	SD
FA	Acute	0.270	0.048	0.356	0.039	0.398	0.031
	Subacute	0.260	0.049	0.349	0.039	0.398	0.036
	Chronic	0.276	0.069	0.341	0.042	0.392	0.037
MD	Acute	0.618	0.125	0.858	0.084	0.837	0.065
	Subacute	0.764	0.103	0.851	0.083	0.831	0.063
	Chronic	1.101	0.237	0.929	0.110	0.851	0.058
AD	Acute	0.793	0.143	1.190	0.075	1.216	0.065
	Subacute	0.968	0.109	1.171	0.072	1.206	0.065
	Chronic	1.394	0.223	1.264	0.102	1.228	0.054
RD	Acute	0.530	0.118	0.692	0.093	0.647	0.067
	Subacute	0.662	0.106	0.691	0.092	0.644	0.067
	Chronic	0.955	0.248	0.761	0.116	0.663	0.064

MD, RD and AD values have been multiplied by 1000.

**Table 3:** ANOVA results

		ANOVA		
		*F*-values	*P*	Effect size
FA	Main effect ROI	*F*(2,32) = 109.33	<0.0001	0.87
	Main effect stage post-stroke	*F*(2,32) = 0.39	0.68	
	Interaction ROI ∗ stage post-stroke	*F*(4,64) = 2.27	0.07	
MD	Main effect ROI	*F*(2,32) = 4.68	0.017	0.23
	Main effect stage post-stroke	*F*(2,32) = 37.92	<0.0001	0.7
	Interaction ROI ∗ stage post-stroke	*F*(4,64) = 40.31	<0.0001	0.72
AD	Main effect ROI	*F*(2,32) = 55.18	<0.0001	0.78
	Main effect stage post-stroke	*F*(2,32) = 56.75	<0.0001	0.78
	Interaction ROI ∗ stage post-stroke	*F*(4,64) = 58.03	<0.0001	0.78
RD	Main effect ROI	*F*(2,32) = 7.44	0.002	0.32
	Main effect stage post-stroke	*F*(2,32) = 28.96	<0.0001	0.64
	Interaction ROI ∗ stage post-stroke	*F*(4,64) = 31.19	<0.0001	0.66

**Table 4:** Summary of *post hoc* results

	*Post hoc* comparison	MD	AD	RD
ROI				
WM lesion	Acute versus subacute	<0.001	<0.0001	<0.001
	Subacute versus chronic	<0.001	<0.0001	<0.001
	Acute versus chronic	<0.0001	<0.0001	<0.0001
pWM	Acute versus subacute	n.s	n.s	n.s
	Subacute versus chronic	<0.001	<0.0001	0.002
	Acute versus chronic	<0.001	<0.0001	0.0001
rWM	Acute versus subacute	n.s	n.s	n.s
	Subacute versus chronic	0.024	0.011	0.047
	Acute versus chronic	n.s	n.s	n.s
Stage post-stroke				
Acute	WM lesion versus pWM	<0.0001	<0.0001	<0.0001
	WM lesion versus rWM	<0.0001	<0.0001	0.0016
	pWM versus rWM	n.s.	n.s.	0.013
Subacute	WM lesion versus pWM	0.011	<0.0001	n.s.
	WM lesion versus rWM	n.s.	<0.0001	n.s.
	pWM versus rWM	n.s.	0.042	0.02
Chronic	WM lesion versus pWM	<0.001	0.006	<0.001
	WM lesion versus rWM	<0.001	0.017	<0.001
	pWM versus rWM	0.0014	n.s.	<0.001

### FA

There was a significant main effect of the ROI, with significantly lower FA values in the WM lesion compared to pWM and rWM and in the pWM compared to the rWM. There was no significant effect of the stage post-stroke or ROI ∗ stage post-stroke interaction.

### MD

The analysis of MD values showed a significant main effect of ROI and of the stage post-stroke. More importantly for the present objective, the analysis revealed a significant ROI ∗ stage post-stroke interaction, indicating that the evolution of the MD metrics at different stage post-stroke varies from a ROI to another.

In the WM lesion, MD values significantly increased from acute and subacute stages and from subacute to chronic stage. Compared to rWM, MD values in the WM lesion were significantly lower at both acute and subacute stages and significantly higher in chronic stage. On the other hand, in the pWM, no MD changes were observed between acute and subacute stages, where the MD values were comparable to the rWM. However, a significant increase of MD was observed in pWM between subacute and chronic stages. A modest albeit significant increase of MD between the subacute and chronic stages was also observed in the rWM. This change was less prominent compared to that observed in pWM (and WM lesion). In fact, MD was significantly higher in pWM compared to rWM in chronic stage. It must be noted that the Cohen’s *d* for the comparison between subacute and chronic stages in rWM (*d* = 0.34) indicates a relatively small effect size, while all the stage comparisons observed in WM lesion and pWM were associated with effect size that exceeded Cohen’s convention for a large effect (*d* = 0.80).

### AD

The analysis of AD values showed a significant main effect of ROI and of stage post-stroke. A significant ROI ∗ stage post-stroke interaction was also observed.

In WM lesion, AD values significantly increased from acute and subacute stages and from subacute to chronic stage. AD values were significantly lower in WM lesion compared to both pWM and rWM at the acute and subacute stages, and they were significantly higher than both pWM and rWM at the chronic stage.

In the pWM, the AD values were stable between acute and subacute stages. AD values in pWM were comparable to rWM in acute stages and slightly but significantly lower (*P* = 0.042, *d* = 0.36) in subacute stage. There was a significant increase of AD in pWM between subacute and chronic stages. An increase of AD between subacute and chronic stages was also observed in rWM. No difference was observed between AD in pWM and in rWM at chronic stage.

### RD

The analysis of RD values showed a significant main effect of ROI (higher RD in both WM lesion and pWM compared to rWM) and of stage post-stroke (higher RD values in subacute compared to acute stages, in chronic compared to subacute stages, and in chronic compared to acute stages) and significant ROI ∗ stage post-stroke interaction.

In WM lesion, RD values significantly increased from acute and subacute stages and from subacute to chronic stage. RD values were significantly lower in WM lesion compared to pWM and rWM at the acute stage, comparable at subacute stage and higher at chronic stage. In pWM, RD values were stable, although higher than rWM, between acute and subacute stages, but they significantly increased between subacute and chronic stages. Subtle but significant increases in increasing of RD between subacute and chronic stages were also observed in rWM (*P* = 0.047, *d* = 0.29). However, RD values were significantly higher in pWM compared to rWM in chronic stage.

### Additional analyses

WM lesion size could differ from a participant to another. Consequently, the size of the pWM, which is based on the dilatation of the WM lesion, could differ as well. In order to control for the WM lesion size, we re-ran the analyses including the size of the WM lesion as a covariate in the model. All the main effects, the interactions and the majority of *post hoc* comparisons reported in the main analyses were still significant after controlling for lesion size. Only few differences from the main analysis were observed, not impacting the main results highlighted in the study. The effects of the ROI at subacute stage (*F*(2,30) = 3.88, *P* = 0.053) and chronic stage (*F*(2,30) = 3.79, *P* = 0.065) on MD values and the effect of stage post-stroke on RD values in the pWM (*F*(2,30) = 2.87, *P* = 0.097) became marginal, most likely due to diminished statistical power. The effect of the stage post-stroke in the rWM for MD (*F*(2,30) = 0.43, *P* = 0.655) and AD (*F*(2,3) = 0.35, *P* = 0.709) and the effects of the ROI on AD values at chronic stage (*F*(2,30) = 0.38, *P* = 0.688) were however not significant.

## Discussion

In the present study, we aimed to explore the pattern of dMRI-based diffusivity metrics in the ipsilesional WM (i.e. in the WM lesion, the pWM and the rWM) after a first left middle cerebral artery ischaemic stroke, in the acute, subacute and chronic stages post-stroke. Overall, our findings suggest that various pathophysiological processes occur at the different time-points following ischaemic stroke, with the infarct territory and perilesional WM affected by ischaemia at different rates and to different extents. The pattern observed in the infarct core in the acute stage is compatible with the early fragmentation of axons and focal axonal swelling. Subsequent rises in AD may reflect the process of removing cellular debris. Large increases in RD and MD values could be a consequence of myelin degradation that occurs at later stage of the disease. This effect would be particularly severe in the site of the ischaemic stroke where decreased blood flow leads to diminished energy supply (triggering myelin damage). Within the pWM area, we observed similar but delayed diffusion changes as in the infarct core, consistent with studies showing diffusion changes occurring first in the infarct and then extending to distal parts of WM tissue. The following paragraphs summarize the diffusion changes occurring in each ROI, and their possible association with pathophysiological mechanisms following ischaemic stroke.

### WM lesion

The overall pattern of diffusion (low FA, MD, AD and RD) observed within the infarct territory at the acute stage suggests that cytotoxic oedema and axonal disintegration are already occurring within the lesion in the first few days after stroke. Specifically, this is compatible with the model of pathophysiological mechanisms of WM injury based on animal studies, according to which a cascade of events initiated by energy reduction due to ischaemia and loss of adenosine triphosphate (ATP) leads to intracellular accumulations of toxic levels of calcium. Ultimately, this results in the degradation of the axonal cytoskeleton and organelles, focal axonal swelling and axonal disintegration. Indeed, we observed low FA, combined with MD decreases, reflecting the structural damages affecting the WM in the ischaemic infarct in the first few days after stroke. While FA and MD were sensitive to early microstructural WM changes, they remained unspecific about the type of change that occurs^7^ and were therefore interpreted in combination with other diffusion metrics. Based on the knowledge of the pathophysiological impact of an ischaemic stroke on WM tissue, we believe that the observed decrease in AD in the lesion site, considered alongside reduced FA and MD, reflects acute axonal disintegration in the infarct territory: as axonal structures degrade due to cell necrosis, cellular debris is created, forming new barriers to diffusion parallel to the axon and reducing FA, MD and AD in the first days after stroke. This was consistent with findings from most animal and human stroke studies.^8–10,12,15,19,23^ While there have been a few reports of normal or even increased FA in the lesion core acutely after stroke,^16,17^ it should be noted that FA also incorporates the magnitude of RD, which can lead to pseudo normal or even elevated FA values hyperacutely. For example, Alegiani *et al*.^16^ and Green *et al*.^17^ reported elevated FA in the infarct territory hyperacutely after stroke (at 8 and <27 h after symptom onset, respectively); whereas low FA has mostly been observed in the later acute window in other studies,^8,10^ including the present one (i.e. between 24 and 72 h). Thus, patient inclusion at various points in the hyperacute and acute stages may result in differences in FA measurements between studies. In accordance with previous evidence,^9,10,16,18,19^ we also found significantly lower RD in the WM lesion at the acute stage (compared to the pWM and rWM), reflecting restricted perpendicular diffusion in this region. Thus, our findings support the idea of cytotoxic oedema occurring at early time-points, decreasing MD and RD due to cellular swelling (‘beading’ of axons) increasing extracellular tortuosity.^6^ Indeed, WM has less collateral blood flow than grey matter, making it particularly vulnerable to rapid cell swelling and tissue oedema after ischaemic stroke.^1^

Then, the overall pattern of diffusion that we observed within the WM lesion in the subacute stage (low FA with MD, AD and RD increases) is compatible with persisting axonal damage, combined with the elimination of cellular debris and a diminution of cell swelling, therefore partially restoring diffusion. Indeed, at the subacute time-point, we found FA values that remained significantly lower in the WM lesion compared to the pWM and rWM, which was consistent with findings from most studies.^9^ This was accompanied by a significant increase in AD, which remained however lower in the infarct territory compared to the pWM and rWM. Low FA, with increasing, but persistently low AD values, most likely reflects the axonal damage within the infarct territory, along with the ongoing process of removing the cellular debris by phagocytic microglia, thereby subtly increasing the diffusion in the longitudinal direction.^40^ Consistent with other studies conducted in the subacute stage e.g.^10^ RD and MD values also increased to levels that were similar to that of the pWM and rWM.^10^ This was attributed to a decrease of cytotoxic oedema, causing increases of the extracellular space restoring diffusion perpendicularly to the axon in the ischaemic territory.^18^

Finally, the pattern of diffusion observed within the WM lesion in the chronic stage (low FA, with elevated MD, RD and AD) suggests that the processes of myelin degradation occur in the later stages of human stroke. Importantly, this may reflect similar mechanisms to those proposed by the animal models of pathophysiological mechanisms of WM injury, in which sustained energy depravation, resulting in prolonged glutamate receptor activation, triggers excitotoxic damage to oligodendrocytes and oligodendrocyte progenitor cells, causing the degradation of myelin sheaths. Indeed, FA remained stable and consistently low in the ischaemic territory from the acute to the chronic stage, reflecting permanent damage to the WM lesion tissue. Then, the findings of chronically elevated MD and RD within the infarct territory compared to the other regions (while FA values remain decreased) are consistent with degradation of myelin sheaths as indicated by increased diffusion perpendicular to the axons in the lesion core.^18^ AD values also continued to increase in the WM lesion between the subacute and chronic stages, and became comparable to the pWM and rWM, indicating little restriction to longitudinal water diffusion in the ischaemic territory in the chronic stage, which most likely reflects the axonal disintegration and the process of the removal of cellular debris hindering water movement that had started in the subacute stage.

### pWM

Within the pWM area, we observed similar but delayed diffusion changes, indicating axonal damage causing acute FA and subacute AD decrease, followed by myelin loss causing chronic increases in MD and RD.

The acute pattern of diffusion in the pWM suggests that some early microstructural changes are visible in the WM surrounding the stroke lesion early after stroke. While the pWM tissue was characterized by lower FA values in the acute stage compared to the rWM, RD was slightly elevated and no significant differences were found for MD and AD (i.e. no clear indication of focal swelling and/or axonal disintegration). These findings indicate a slight diminution of diffusion anisotropy, which may suggest an early loss of structural integrity (e.g. due to focal axonal damage) in the WM surrounding the infarct site. This was compatible with results of Groisser *et al*.^13^ and Shaheen *et al*.^2^ Both studies found a significant loss of FA, but no change in other parameters in the peri- and ipsilesional corticospinal tract in patients who had suffered acute ischaemic stroke in the middle cerebral artery territory. Similarly, Doughty *et al*.^14^ found lower mean FA in patients with motor impairment in the ipsilesional corticospinal tract within 80 h after ischaemic stroke onset in the areas that were the closest to the original infarct.

Then, the pattern of reduced FA and AD observed in the pWM in the subacute stage suggests that signs of axonal disintegration become visible subacutely in the pWM (versus acutely in the WM lesion). Indeed, while FA values stayed low, AD values were lower in the pWM compared to rWM, consistently with findings from Liu *et al*.,^21^ who had found a significant reduction of AD in the ipsilesional corticospinal tract above the primary lesion in the first week after subcortical infarction. This reflects reduced diffusion longitudinally, suggesting that some axonal damages have occurred within the pWM.

Then, our findings regarding the pWM in the chronic stage indicate low FA with increases in AD, RD and MD and are consistent with persisting damage to WM bordering the lesion, which includes some extent of myelin damage. Such diffusion changes have been found in most studies on human stroke. For instance, in Buffon *et al*.,^8^ low FA with increased MD was noted in the ipsilesional hemisphere at 6 months following middle cerebral artery infarction. Liu *et al*.^21^ found increases in AD in the ipsilesional corticospinal tract above the primary lesion between the first week after subcortical infarction and a 12-week follow-up. In Umarova *et al*.,^20^ patients displayed longitudinal white matter alterations that included perilesionally reduced FA and increased RD, AD and MD in the chronic stage. This pattern of diffusion, with low FA and increased RD and MD, indicates increased perpendicular (and overall) diffusion in the pWM tissue, reflecting myelin disintegration processes occurring at the chronic stage that, while less pronounced, may be comparable to those occurring in the WM lesion.

Overall, our findings are consistent with several studies showing that diffusion changes occur first in the infarct and then extend to distal parts of WM fibres.^13,41^ This is consistent with the idea that the further away from the infarct site, the longer it takes for damage to occur. According to previous work, these more subtle changes may be explained by the anterograde or retrograde degeneration of fibres directly damaged by the lesion (i.e. Wallerian degeneration).^42^ Specifically, in addition to the changes in the infarct itself, the infarct lesion may cut off the nutritional supply in the surrounding WM, leading to degeneration of the nerve fibres, including axonal and myelin sheath disintegration.

### rWM

Our findings regarding the rWM area support the idea that this region remains overall unaffected by the changes associated with ischaemia at the different stages. Thus, our findings are consistent with studies suggesting that the visible microstructural damages remain constrained to the WM tissue that is the closest to the infarct territory, with no involvement of the WM further from the infarct site. For instance, Doughty *et al*.^14^ found lower mean FA in patients with motor impairment in the ipsilesional corticospinal tract within 80 h after ischaemic stroke onset, but this early WM degeneration was mostly visible in the areas that were the closest to the original infarct. Similarly, concerning changes from the acute to the chronic stage of stroke, Umarova *et al*.^20^ showed that degenerative changes associated with FA reduction and MD or AD increases remained constrained to the perilesional region. While there have been some previous reports of microstructural changes in the ipsilesional WM following stroke,^18^ it should be noted that the selected ROIs appeared to also encompass the lesion and/or the perilesional sites. This may help to explain the discrepancy between the results and further reflects the importance of delimiting ROIs by their proximity to the lesion site.

### Limitations

Future studies could consider replicating these findings in larger datasets. This could allow the assessment of the association between the evolution of diffusion metrics and other variables, such as demographical (e.g. age and sex) and behavioural data. Although the aetiology of the stroke was controlled (only first left middle cerebral artery stroke was included in the study), lesion size and location could differ from one subject to another. The additional analysis controlling for WM lesion size confirmed the main results presented in the article, indicating that they cannot be entirely ascribed to lesion size differences. Considering that the lesions could have different locations in different participant, it is highly possible that the mean dMRI metrics extracted in the present study using a voxel-based ROI approach belong to different fibre tracts in different individuals. This is an intrinsic limitation of the approach. Complementary studies using fibre tracking approaches should be conducted for verifying whether dMRI tracts belonging to different tracts have different profiles.

## Conclusion

The present study reports detailed and longitudinal diffusion changes in lesional, perilesional and remaining ipsilesional WM tissue. Although an association between diffusion metrics and pathophysiological mechanisms cannot be directly established, our results show a pattern that is consistent with different mechanisms that are at play at different stages following stroke. Generally, our findings also indicate that considering FA alone, or FA and MD, only gives partial information about the pathophysiological changes occurring after stroke. Indeed, while the examination of FA values suggests a disruption of structural integrity at all time-points in the infarct and perilesional territory, only the combination of FA with other diffusivity metrics, combined with the knowledge of pathophysiological processes following stroke, can give us more information about the mechanisms that occur at different time-points (e.g. focal swelling, axonal damage and myelin loss).

## Data Availability

The data that support the findings of this study (the python script for creating the ROIs, dMRI-based FA, MD, AD and RD maps, ROIs masks for each patient and spreadsheet with individual metrics values at each time-points for each ROI) are available on request from the corresponding author. The raw data are not publicly available because participants did not consent sharing their data.
